# Computational research of Belnacasan and new Caspase-1 inhibitor on cerebral ischemia reperfusion injury

**DOI:** 10.18632/aging.203907

**Published:** 2022-02-22

**Authors:** Hui Li, Zhen Guo, Jun Chen, Zhishan Du, Han Lu, Zhenhua Wang, Jianxin Xi, Yang Bai

**Affiliations:** 1 Department of Neurosurgery, The First Hospital of Jilin University, Changchun, China; 2 Department of Neurology, The First Hospital of Jilin University, Changchun, China; 3 Clinical College, Jilin University, Changchun, China

**Keywords:** cerebral ischemia-reperfusion injury (CIRI), Caspase- 1, inhibitor, inflammation, pyroptosis

## Abstract

Cerebral ischemia-reperfusion injury is one of the most severe diseases in terms of mortality and disability, which seriously threatens human life and health. In clinical treatment, drug thrombolysis or mechanical interventional thrombolysis are used to quickly restore the blood supply of ischemic brain tissue. But with the rapid recovery of blood flow, complex pathophysiological processes such as oxidative stress and inflammation will further aggravate brain tissue damage, namely cerebral ischemia-reperfusion injury, for which there is no effective treatment. Recent studies have shown that the medical community has paid the role of inflammation and pyroptosis in cerebral ischemia-reperfusion injury more and more attention. And Caspase-1 was found to play a vital role in regulating inflammation pathways and pyroptosis in many inflammation-associated diseases, especially in cerebral ischemia-reperfusion injury. Not only that, Caspase-1 inhibitors have been shown to reduce the damage of cerebral ischemia-reperfusion injury by inhibiting inflammation and pyroptosis. And the Caspase-1 inhibitor, Belnacasan, has been proved to modify the active site of Caspase-1 and lead to the blocking of Caspase-1, thus correlating with tissue protection of inflammatory diseases in animal models. Therefore, it’s essential to screen and design potential Caspase-1 inhibitors to reduce cerebral ischemia-reperfusion injury and protect brain function by reducing inflammation and pyroptosis, which provides a new idea for clinical treatment of the cerebral ischemia-reperfusion injury. This study applied a group of computer-aided technology, such as Discovery Studio 4.5, Schrodinger, and PyMol, to screen and assess potential Caspase-1 inhibitors. Moreover, the ADME (absorption, distribution, metabolism, excretion) and TOPKAT (Toxicity Prediction by Computer Assisted Technology) molecules of Discovery Studio 4.5 were conducted to evaluate molecules' pharmacological and toxicological features. Then, precise molecular docking was applied to assess the binding mechanism and affinity between Caspase-1 and selected compounds. Besides, molecular dynamics simulations were performed to determine the stability of ligand-receptor complexes in the natural environment. In summary, this study lists promising drug candidates and their pharmacological properties, promoting the development of Caspase-1 inhibitors and deepening the understanding of the interaction between inhibitors and Caspase-1.

## INTRODUCTION

Cerebral ischemia-reperfusion injury (CIRI) is one of the most severe diseases in terms of mortality and disability, which seriously threatens human life and health [1]. In clinical treatment, the blood supply of ischemic brain tissue is rapidly restored mainly through drug thrombolytic or mechanical interventional thrombectomy [2]. But with the rapid recovery of blood flow, complex pathophysiological processes such as oxidative stress and inflammation will further aggravate brain tissue damage, namely CIRI, for which there is no effective treatment. The pathological mechanism of CIRI is very complex, and currently, known mechanisms involved in this process include inflammatory response, autophagy, mitochondrial dysfunction, calcium overload, etc. Several studies have shown that inflammatory response and pyroptosis play an essential role in the pathological process of CIRI and are closely related to the mechanisms mentioned above [3].

In the early stage of cerebral ischemia, due to slow blood flow, neutrophils attach to the endothelial cells of the ischemic vessels, and acute inflammatory responses begin. After cerebral ischemia-reperfusion, the bloodstream carries other exogenous inflammatory cells to reach the ischemic brain tissue through the blood-brain barrier, blocking micro-vessels, resulting in secondary hypoperfusion and the release of a large number of oxygen-free radicals [4]. Meanwhile, in the central system, microglia are induced and activated by astrocytes to proliferate and produce various inflammatory mediators. These inflammatory mediators activate endothelial cells to produce multiple tissue factors, increase the toxicity of excitatory amino acids, and promote the release of nitric oxide and oxygen-free radicals [5]. These substances further lead to the activation of NF-κB(nuclear factor kappa-B), JNK2/STAT3(The Janus kinase/signal transducer and activator of transcription ions) and other inflammatory signal transduction pathways, and promote the assembly of NLRP3 (Nod-like receptor Pyrin domain Three) and other inflammasomes, thereby activating Caspase-1 (Cysteine-dependent aspartate-specific protease 1). Activated Caspase-1 induces pyroptosis of cells, enlarges inflammatory response, and aggravates the CIRI. Therefore, Caspase-1 is a critical protein that aggravates the CIRI, and inhibition of its expression and activation can simultaneously Inhibit the expression of proteins associated with both downstream inflammation and pyroptosis pathways, thereby alleviating CIRI.

Caspases are a protease family with similar amino acid sequences and structure, which cleave polypeptide substrates containing aspartic acid with Cys-containing active sites, consequently regulating apoptosis, inflammation, differentiation, and proliferation [6]. The caspase family can be divided into apoptosis-related and inflammation-related categories according to different functions. Caspase-3, 10 mainly mediate cell death, while Caspase-1, 4, 5, and 11 are crucial inflammation and innate immune response mediators. Caspase-1, also called IL-1-converting enzyme (ICE), contains a caspase recruitment domain (CARD), a central p20 subunit domain, and a p10 subunit domain when existing as an inactive zymogen in the cytoplasm. Under normal conditions, pro-Caspase-1 exists in the cytoplasm of cells as an inactive proenzyme. After being activated, the CARD is removed, and subunits are separated [7].

Caspase-1 is activated primarily by inflammasome NLRP3 (Nod-like receptor Pyrin domain Three). Inflammasome NLRP3 plays an immunological role in the cytoplasm. It is contained by NLRP3, ASC (apoptosis-associated speck-like protein containing a CARD), and pro-Caspase-1 [8]. NLRP3 receptor consists of PYD (pyrin domain), NOD (nucleotide-binding and oligomerization domain), and LRR (leucine-rich repeat) [9]. The PYD domain mediates the interaction with the thermo protein domain of ASC, leading to inflammasome assembly. NOD domain has ATPase activity, which can promote the auto-oligomerization of NLRP3. NLRP3 can be activated by various stimuli, such as reactive oxygen species and ATP [10–13]. The activated NLRP3 oligomerizes itself and binds to the PYD domain of ASC. ASC recruits pro-Caspase-1 through the CARD domain to form NLRP3 inflammasome. NLRP3 inflammasome splashes pro-Caspase-1 into active Caspase-1 (P20), which splashes inflammatory cytokines IL-1 β(Interleukin-1β) and IL-18 (Interleukin-18) precursors into active forms, thereby initiating various downstream signaling pathways that trigger inflammatory responses [14–16]. In addition, the latest studies found that GSDMD (GasderminD protein) is the co-acting substrate of Caspase-1, Caspase-11, and caspase-4, 5, and is the effector protein that causes cell pyroptosis [17]. The activated inflammatory Caspase cleaved GSDMD, relieved its self-inhibition, and released active N-terminal residues. The N-terminal residues then bind to the membrane, forming pores of 10-15nm (nanometer) in the membrane, resulting in changes in cell permeability and the release of many mature pro-inflammatory factors. In general, the above eventually leads to cell pyroptosis and a cascade of inflammatory responses. Moreover, Caspase-1 can also damage the blood-brain barrier during cerebral ischemia-reperfusion [18]. Therefore, Caspase-1 plays a vital role in CIRI and is expected to become a target of cerebrovascular protection in the future [19].

Studies on Caspase-1’s inhibitors have attracted a lot of attention. Several inhibitors of Caspase-1 have been reported, such as Belnacasan (VX-765), AC-YVAD-CMK, AC-DEVD-CHO, Q-VD-Oph, Z-VAD-FMK, Mulberroside A, Chelidonic acid, etc. [20–24]. Among these, Belnacasan is in phase II clinical trials and is the most studied inhibitor known [25–27]. Belnacasan, the orally absorbed prodrug of VRT-043198, can be quickly converted into VRT-043198 *in vivo* and covalently modified the catalytic cysteine residue in the active site of Caspase-1, thus leading to Caspase-1 blocking [26]. And inhibition of Caspase-1 correlates with tissue protection of inflammatory diseases in animal models [27, 28]. Moreover, studies have found that Belnacasan treatment during the reperfusion process can significantly reduce infarct size and the negative impact of ventricular function on rats significantly [29]. Early treatment of Belnacasan may prevent the onset of cognitive deficits and brain inflammation in Alzheimer's disease [30]. But Belnacasan has not yet been used in the clinic. Although the research on Caspase-1 inhibitors has achieved excellent results, the inhibitors are still in the research stage. Therefore, it is of great significance to develop new inhibitors and explore the mechanism of interaction between inhibitors and Caspase-1 to improve CIRI.

With drug research development, natural products play an increasingly important role in molecular biological research and drug exploration. In this study, we chose Belnacasan as the reference drug. Firstly, we conducted a virtual screening on the NP (Natural Products) Database in the ZINC database to explore potential Caspase-1 inhibitors. Secondly, the ADME (absorption, distribution, metabolism, excretion) and TOPKAT (Toxicity Prediction by Computer-Assisted Technology) modules of Discovery Studio 4.5 software (DS 4.5) were carried out to inspect pharmacological and toxicological features. Then, we performed molecular docking with DS 4.5, PyMol, and Schrodinger to evaluate the interactions between the selected compounds and Caspase-1. Besides, the pharmacophore prediction of compounds was carried out. In the end, we analyzed the stability of binding interaction through molecular dynamics simulation. To summarize, this study lists a suite of drug candidates and their pharmacological properties, which could promote the development of Caspase-1 inhibitors and deepen the understanding of the interaction between inhibitors and Caspase-1.

## MATERIALS AND METHODS

### Docking software and ligand database

Discovery Studio is a molecular modeling and simulation environment software for protein structure, function research, and drug discovery [31]. In this study, Libdock, ADME, and TOPKAT modules of Discovery Studio 4.5 software (DS 4.5, Accelrys, Inc) were used for screening potential Caspase-1 inhibitors, while the CDOCKER module was employed for molecular docking. Additionally, other structure-based virtual screening software, such as Schrodinger and PyMol, were also applied to further display the binding interactions of ligands and Caspase-1. Moreover, ZINC Database, a free virtual screening database of commercial compounds, was selected as the ligand database. It is provided and maintained by the Irwin and Shoichet Laboratories in the Department of Medicinal Chemistry at the University of California, San Francisco (UCSF). And this database contains more than 750 million compounds available for purchase, of which 230 million are provided with 3D files. In this study, a total of 17799 3D molecular files of natural, named, and purchasable molecules were downloaded from the ZINC15 database for virtual screening Caspase-1 inhibitors.

### Structure-based virtual screening using libdock

**Figure 1:**
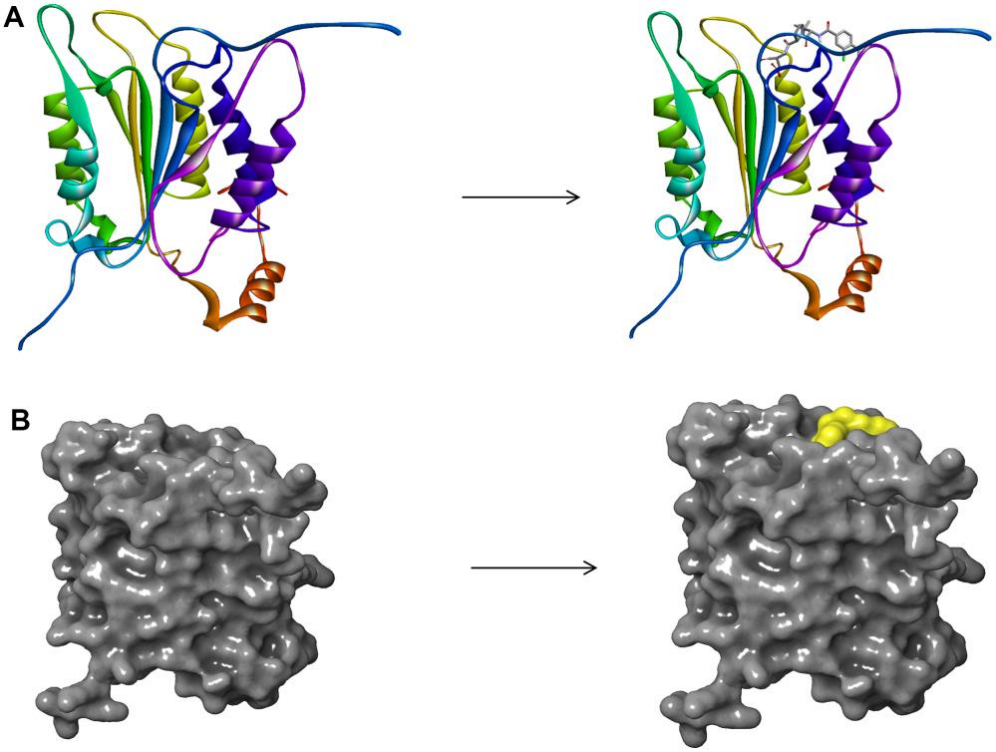
(**A**) The molecular structure of Caspase-1 and the complex structure of Caspase-1 with Belnacasan. Initial molecular structure was shown. (**B**) The molecular structure of Caspase-1 and the complex structure of Caspase-1 with Belnacasan. The surface of the complex was added, green for Belnacasan and gray for Caspase-1.

Based on the Libdock module of DS 4.5, Caspase-1 and Belnacasan (VX-765) 's binding position served as a docking site to screen novel Caspase-1 inhibitors. Libdock was a rigid-based docking program, which employed polar and nonpolar probes and grids placed in binding sites to calculate hotspots of proteins. Afterward, the hot spots were used to arrange ligands to form well interactions. Then, Ligand minimization was carried out by Smart Minimiser algorithm and CHARMm forcefield (Cambridge, MA, USA). In the end, all the selected ligands were ranked based on the ligand score. The crystal structure of Caspase-1 in complex with Belnacasan (1.94 Å) was downloaded from protein data bank (PDB) and imported into DS 4.5 for Libdock. The 3D structures of Caspase-1 and the Belnacasan- Caspase-1 complex are displayed in Figure 1. Some procedures such as minimization of energy, hydrogenation, ionization, protonation, and removing crystal water and other heteroatoms were employed during protein preparation [32]. Besides, the energy minimization was also carried out by the Smart Minimiser algorithm and CHARMm forcefield.

### Adsorption, distribution, metabolism, excretion (ADME), and toxicity prediction

The absorption, distribution, metabolism, and excretion properties of selected compounds were calculated through the ADME module of DS 4.5, such as aqueous solubility, plasma protein binding (PPB) level, blood-brain barrier (BBB) penetration, cytochrome P450 2D6 (CYP2D6) inhibition, hepatotoxicity, human intestinal absorption. And the TOPKAT (Toxicity Prediction by Computer Assisted Technology) module of DS 4.5 was also applied to predict toxicity properties of selected compounds, including rodent carcinogenicity, Ames mutagenicity (AMES), and developmental toxicity potential (DTP). All these pharmacological and toxicological properties were considered when selecting drug candidates for Caspase-1.

### Molecular docking and pharmacological analysis

The CDOCKER module of DS 4.5 was employed for precise molecular docking between Caspase-1 and selected compounds based on CHARMm forcefield. The ligands can flex during the docking process while the receptor remains rigid. The CDOCKER interaction energy and absolute energy of the complexes, which stand for ligand binding affinity, were assessed. In general, since fixed water molecules may affect the receptor-ligand complex formation, crystalline water molecules were deleted during rigid and semi-flexible docking processes. After the water molecules were removed, hydrogen atoms were added to the protein. Then, the original compound Belnacasan was separated from the binding site and then re-docked into the crystal structure of Caspase-1 to testify the dependability of the combination mode. Subsequently, the CHARMm36 forcefield was employed for both receptors and ligands. The region within radius 13 Å served as the binding site sphere of Caspase-1 from the geometric centroid of the ligand Belnacasan. The ligand is gradually bound to the residues in the binding site sphere in the docking procedure. Then, the structure of identified hits was docked into the binding pocket of Caspase-1. During the CDOCKER process, each ligand generated ten docking postures, and the best posture was chosen based on high docking scores and reasonable docking orientations. According to the CDOCKER interaction energy and absolute energy, different postures of each selected molecule were generated and evaluated. The binding of the best posture for selected molecules and protein was displayed further by Schrodinger and PyMol software.

In addition, we also revealed the pharmacophore of the compound using the 3D-QSAR pharmacophore generation module of DS 4.5. Only those with energy lower than 10 kcal/mol can be retained, and up to 255 confirmations can be generated per molecule. Moreover, pharmacophore’s predictions of selected molecules and Belnacasan were also supplemented with a comparative analysis on Schrodinger.

### Molecular dynamic simulation

After molecular docking, the best conformation of each ligand-receptor complex was selected for molecules dynamic simulation. The compound- Caspase-1 complex was put in an orthorhombic box and solvated using an explicit periodic boundary solvated water model. Then, sodium chloride was added to the system with an ionic strength of 0.145 to simulate the physiological environment. Afterward, the system was put in a CHARMm forcefield and relaxed by minimizing (500 steps of conjugate gradient and 500 steps of steepest descent) with the final RMS (root mean square) gradient of 0.289. Then, the system temperature slowly rose from the initial temperature of 50 K to the target temperature of 300 K with the time step of 2 fs. And equilibration simulation took 5 ps. Molecular dynamics simulation (production) was carried out for 80 ps in a time step of 1 fs. The whole simulation process was completed at normal atmospheric pressure, and a relatively constant temperature of nearly 300 K. The particle mesh Ewald (PME) algorithm was used to calculate the long-range electrostatic. The linear constraint solution (LINCS) algorithm was used to fix all the hydrogen-involved bonds. Based on the initial complex settings, the trajectory protocol module of DS 4.5 was used to determine the trajectory for potential energy, structural characteristic, root-mean-square deviation (RMSD).

## RESULTS

### Virtual screening of natural products database against inhibitor of Caspase-1

The binding pocket of the Belnacasan- Caspase-1 complex was an important regulatory site and was selected as a reference site for selecting potential inhibitors of Caspase-1. What’s more, 17799 natural, named, and purchasable molecules were chosen from the ZINC15 database. Belnacasan served as a reference molecule to estimate all the selected compounds' stability and binding affinity. And the higher the Libdock score of compounds, the better docking activity will be. Thus, based on the Libdock algorithm, 9909 compounds had stable interactions with Caspase-1, and 1179 compounds were identified to have higher Libdock scores than Belnacasan (108.4). The top 20 ranked compounds were listed in Table 1 based on the Libdock score.

**Table 1:** Top 20 ranked compounds with higher libdock scores than Belnacasan.

**Number**	**Compounds**	**Libdock score**	**Number**	**Compounds**	**Libdock score**
1	ZINC000085544839	190.486	11	ZINC000095620524	162.546
2	ZINC000062238222	182.783	12	ZINC000004096894	162.449
3	ZINC000085545908	182.519	13	ZINC000004099069	160.577
4	ZINC000085826837	178.487	14	ZINC000100634116	158.968
5	ZINC000085541163	174.756	15	ZINC000042805482	158.94
6	ZINC000008552069	168.116	16	ZINC000100590636	158.043
7	ZINC000004096889	166.979	17	ZINC000004096892	157.545
8	ZINC000014712793	166.502	18	ZINC000004096893	157.141
9	ZINC000004099068	165.785	19	ZINC000014233122	156.81
10	ZINC000013513540	164.163	20	ZINC000040165309	156.334

### Adsorption, distribution, metabolism, excretion (ADME) and toxicity prediction

The ADME properties of Belnacasan and all the selected ligands were predicted using the ADMET module of DS 4.5, including aqueous solubility, cytochrome P450 2D6 (CYP2D6) binding, brain/blood barrier (BBB), hepatotoxicity, human intestinal absorption level, and Plasma Protein Binding properties (PPB). As shown in Table 2, PPB showed that all compounds were weakly bound with plasma protein, including Belnacasan. 11 compounds and Belnacasan were soluble in water. 15 compounds and Belnacasan had an inferior intestinal absorption level, and 5 compounds had a poor absorption level, indicating a potential administration method. Moreover, none of the compounds were predicted to be inhibitors of CYP2D6, an essential enzyme in drug metabolism. In terms of hepatotoxicity, 10 compounds were expected to be non-toxic compared to Belnacasan (toxic).

**Table 2:** ADME (adsorption, distribution, metabolism, excretion) properties of compounds.

**Number**	**Compounds**	**Solubility Level^a^**	**BBB level^b^**	**CYP2D6^c^**	**Hepatotoxicity^d^**	**Absorption Level^e^**	**PPB Level^f^**
1	ZINC000085544839	3	4	0	1	3	0
2	ZINC000062238222	3	4	0	1	3	0
3	ZINC000085545908	3	4	0	0	3	0
4	ZINC000085826837	2	4	0	0	2	0
5	ZINC000085541163	2	4	0	0	2	0
6	ZINC000008552069	4	4	0	1	3	0
7	ZINC000004096889	2	4	0	1	3	0
8	ZINC000014712793	4	4	0	0	3	0
9	ZINC000004099068	3	4	0	0	3	0
10	ZINC000013513540	4	4	0	1	3	0
11	ZINC000095620524	4	4	0	1	3	0
12	ZINC000004096894	2	4	0	1	3	0
13	ZINC000004099069	3	4	0	0	3	0
14	ZINC000100634116	3	4	0	0	2	0
15	ZINC000042805482	2	4	0	0	2	0
16	ZINC000100590636	3	4	0	0	2	0
17	ZINC000004096892	2	4	0	1	3	0
18	ZINC000004096893	2	4	0	1	3	0
19	ZINC000014233122	4	4	0	0	3	0
20	ZINC000040165309	2	4	0	0	3	0
21	Belnacasan	4	4	0	1	3	0

^a^Aqueous-solubility level: 0 (extremely low); 1 (very low, but possible); 2 (low); 3 (good); 4 (very good).

^b^Blood Brain Barrier level: 0 (Very high penetrant); 1 (High); 2 (Medium); 3 (Low); 4 (Undefined).

^c^Cytochrome P450 2D6 level: 0 (Non-inhibitor); 1 (Inhibitor).

^d^Hepatotoxicity: 0 (Nontoxic); 1 (Toxic).

^e^Human-intestinal absorption level: 0 (good); 1 (moderate); 2 (poor); 3 (very poor).

^f^Plasma Protein Binding: 0 (Absorbent weak); 1 (Absorbent strong).

**Figure 2:**
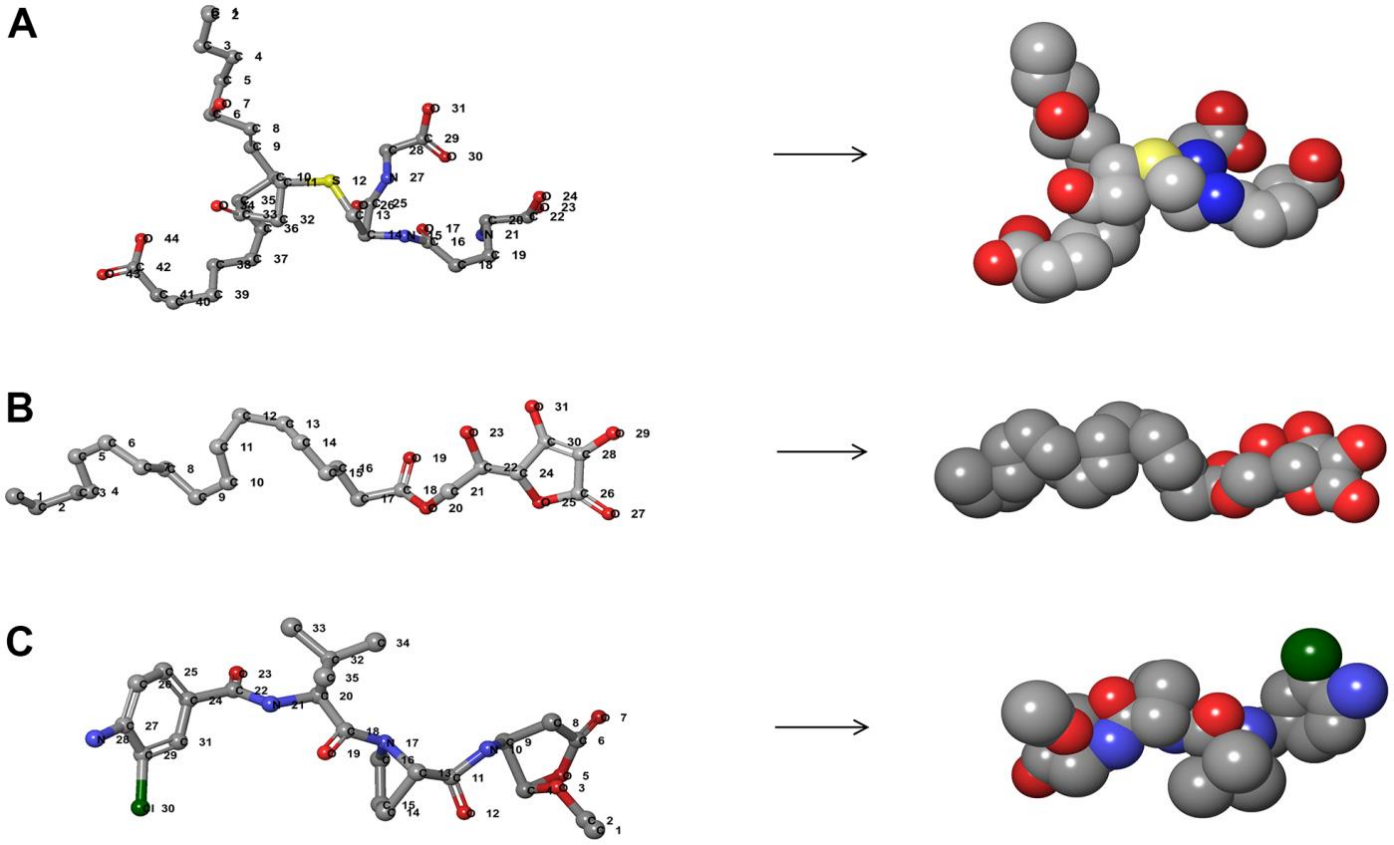
**The 3D structures of Belnacasan and novel compounds selected from virtual screening by Schrodinger.** (**A**) ZINC000004099068; (**B**) ZINC000100634116; (**C**) Belnacasan.

**Figure 3:**
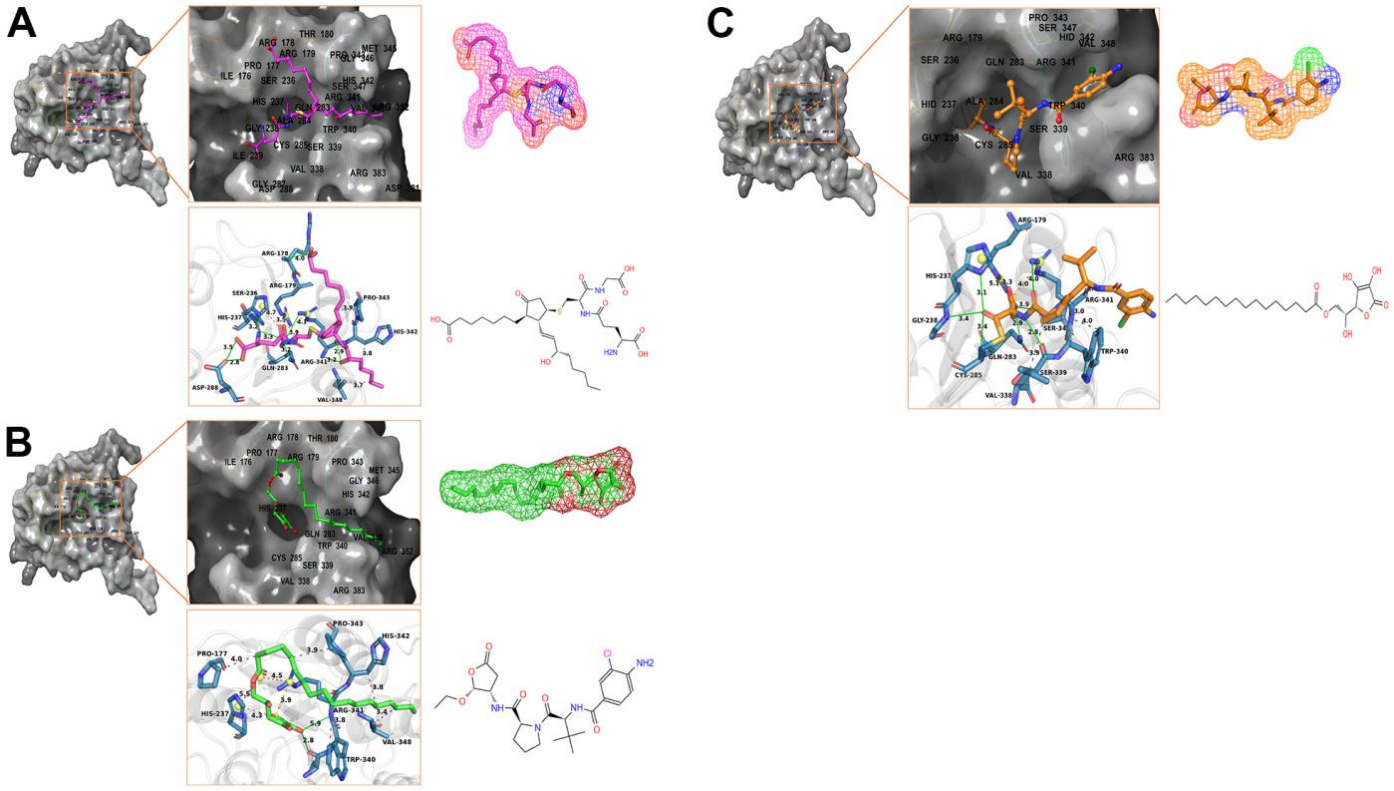
**Schematic drawing of interactions between ligands and Caspase-1 by Schrodinger and Pymol.** Hydrogen bonds, Salt Bridges, and Hydrophobic bonds are shown in green, orange, and gray. The amino acids in the binding pocket; the amino acids that form the bonds; the bond lengths are labeled. The bonding amino acids are in the form of dark blue sticks. (**A**) ZINC000004099068- Caspase-1 complex: Structures and net electron cloud structures of ZINC000004099068 are shown in purple sticks. (**B**) ZINC000100634116- Caspase-1 complex: Structures and net electron cloud structures of ZINC000100634116 are shown in green sticks. (**C**) Belnacasan - Caspase-1 complex: Structures and net electron cloud structures of Belnacasan are shown in orange sticks.

From the perspective of safety, we deeply examined different toxicity such as developmental toxicity potential (DTP), rodent carcinogenicity, and Ames mutagenicity (AMES) properties of compounds and Belnacasan using TOPKAT module of DS 4.5 (Table 3). The result indicated twelve compounds were non-mutagen. Five compounds were predicted to be non-carcinogens, and seven had no developmental toxicity potential. By analyzing Tables 2, 3, it can be seen that ZINC000004099068 and ZINC000100634116 were not inhibitors of CYP2D6, without hepatotoxicity and Ames mutagenicity. What’s more, these two molecules were predicted with no rodent carcinogenicity. According to Figures 2, 3, these two compounds also had structural similarities with Belnacasan, such as ring structure and reactive oxygen atoms, indicating that they might have similar functions. Therefore, ZINC000004099068 and ZINC000100634116 were predicted to be potential candidates and selected for subsequent studies.

**Table 3:** Toxicities of compounds.

**Number**	**Compounds**	**Mouse NTP^a^**		**Rat NTP^a^**		**AMES^b^**	**DTP^c^**
		**Female**	**Male**	**Female**	**Male**		
1	ZINC000085544839	0.4514	0.2498	0.3789	0.4380	0.3409	0.5168
2	ZINC000062238222	0.3846	0.1513	0.3595	0.3850	0.3428	0.4983
3	ZINC000085545908	0.1665	0.0163	0.1748	0.3385	0.0000	0.4320
4	ZINC000085826837	0.4438	0.3649	0.3071	0.1583	0.0968	0.8155
5	ZINC000085541163	0.4438	0.3649	0.3071	0.1583	0.0968	0.8155
6	ZINC000008552069	0.4514	0.2498	0.3789	0.4380	0.3409	0.5168
7	ZINC000004096889	0.5342	0.4427	0.2882	0.4783	0.4573	0.5295
8	ZINC000014712793	0.0466	0.3500	0.0404	0.0599	0.1598	0.7223
9	ZINC000004099068	0.2678	0.0011	0.1309	0.2903	0.0138	0.3672
10	ZINC000013513540	0.1048	0.5246	0.0817	0.0774	0.4212	0.6909
11	ZINC000095620524	0.3922	0.1799	0.2901	0.1638	0.4137	0.6306
12	ZINC000004096894	0.5590	0.4728	0.2861	0.4732	0.4396	0.5218
13	ZINC000004099069	0.2678	0.0011	0.1309	0.2903	0.0138	0.3672
14	ZINC000100634116	0.5335	0.5179	0.1415	0.2967	0.0000	0.5242
15	ZINC000042805482	0.4438	0.3649	0.3071	0.1583	0.0968	0.8155
16	ZINC000100590636	0.5335	0.5179	0.1415	0.2967	0.0000	0.5242
17	ZINC000004096892	0.5590	0.4728	0.2861	0.4732	0.4396	0.5218
18	ZINC000004096893	0.5590	0.4728	0.2861	0.4732	0.4396	0.5218
19	ZINC000014233122	0.4109	0.0619	0.0837	0.3412	0.0000	0.4819
20	ZINC000040165309	0.3877	0.3185	0.1363	0.3555	0.0000	0.3438
21	Belnacasan	0.4701	0.0170	0.3563	0.4151	0.0340	0.3771

^a^<0.3 (Non-Carcinogen); >0.7 (Carcinogen).

^b^<0.3 (Non-Mutagen); >0.7 (Mutagen).

^c^<0.3 (Non-Toxic); >0.7 (Toxic).

### Ligand binding and pharmacophore analysis

The Root Mean Square Deviation (RMSD) between the crystal structure of the complex and the docked pose was 0.6 Å, which proved the high reliability of the CDOCKER module. ZINC000004099068 and ZINC000100634116 were precisely docked into the binding pocket of Caspase-1 to analyze ligand-binding mechanisms using the DS 4.5’s CDOCKER module. The lower the interaction energy, the higher the stability and affinity of the ligand and protein binding. As is shown in Table 4, the interaction energy of ZINC000004099068 and ZINC000100634116 were at a lower level, which indicates that they might have a higher binding affinity with Caspase-1. At the same time, the control drug Belnacasan failed when it was docked with Caspase-1 using CDOCKER, which also showed the binding affinity and stability of the Belnacasan and Caspase-1 were slightly poor. In addition, we further compared the absolute energy. The lower the absolute energy, the more stable the complex of ligand and protein. Results showed that the absolute energy of these two small molecules was lower than Belnacasan, which was consistent with the result of the interaction energy.

**Table 4:** CDOCKER interaction energy and absolute energy of compounds with Caspase-1.

**Compounds**	**CDOCKER Interaction energy (Kcal/mol)**	**Absolute energy (Kcal/mol)**
ZINC000004099068	—68.9253	46.3042
ZINC000100634116	—46.5567	38.655
Belnacasan	—	86.1462

**Figure 4:**
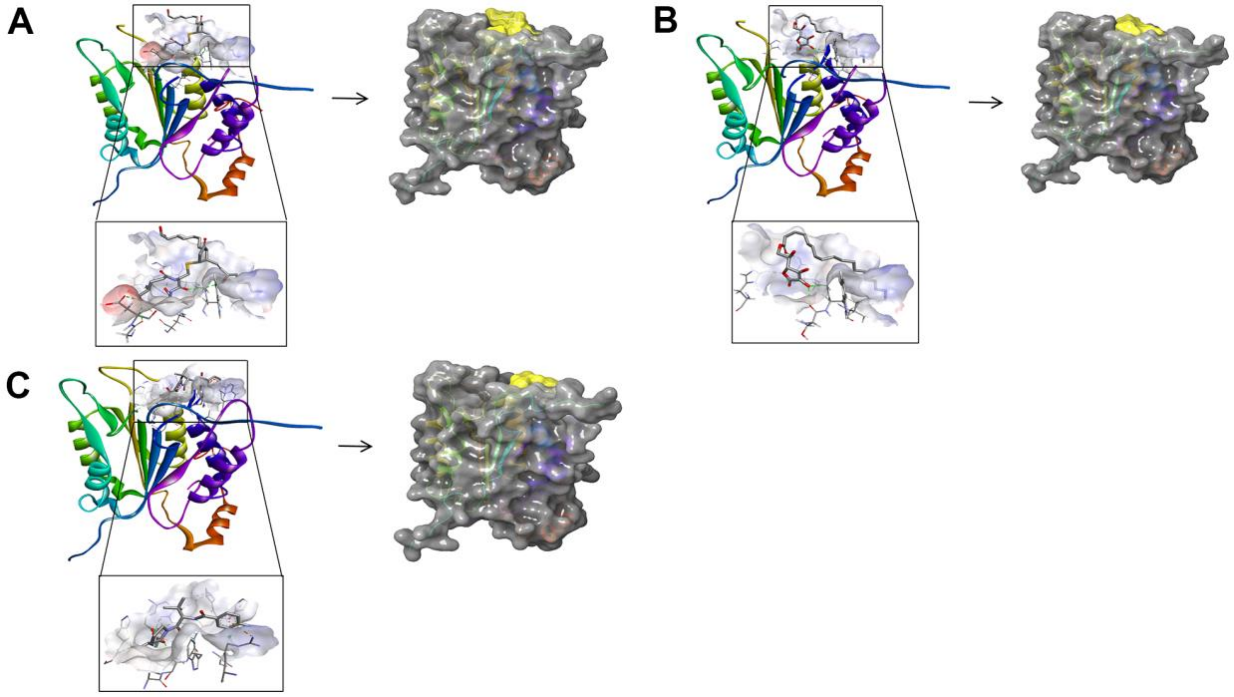
(**A**) ZINC000004099068- Caspase-1 complex. Schematic drawing of interactions between ligands and Caspase-1 by DS 4.5 and Schrodinger. The surface of the binding area was added; blue represented positive charge; red represented negative charge; ligands were shown in sticks; the structure around the ligand-receptor junction was shown in thinner sticks. In addition, the surface of the complex was added, green for ligands and gray for Caspase-1. (**B**) ZINC000100634116- Caspase-1 complex. Schematic drawing of interactions between ligands and Caspase-1 by DS 4.5 and Schrodinger. The surface of the binding area was added; blue represented positive charge; red represented negative charge; ligands were shown in sticks; the structure around the ligand-receptor junction was shown in thinner sticks. In addition, the surface of the complex was added, green for ligands and gray for Caspase-1. (**C**) Belnacasan - Caspase-1 complex. Schematic drawing of interactions between ligands and Caspase-1 by DS 4.5 and Schrodinger. The surface of the binding area was added; blue represented positive charge; red represented negative charge; ligands were shown in sticks; the structure around the ligand-receptor junction was shown in thinner sticks. In addition, the surface of the complex was added, green for ligands and gray for Caspase-1.

**Figure 5:**
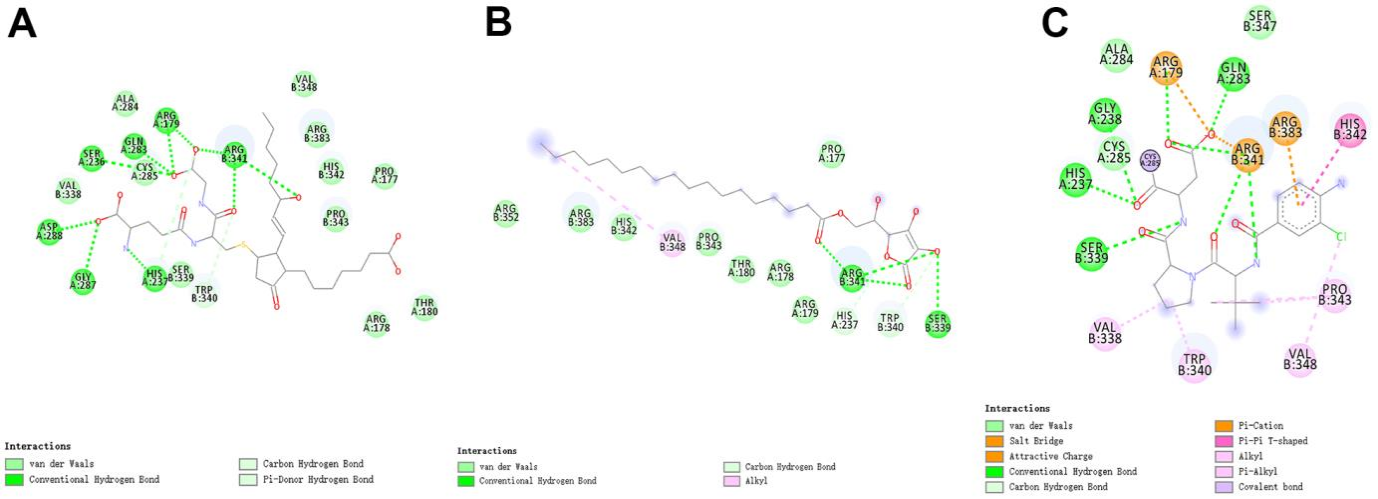
The intermolecular interaction in the binding pockets by DS 4.5 of the predicted binding modes of (**A**) ZINC000004099068 to Caspase-1; (**B**) ZINC000100634116 to Caspase-1, (**C**) Belnacasan to Caspase-1.

**Figure 6:**
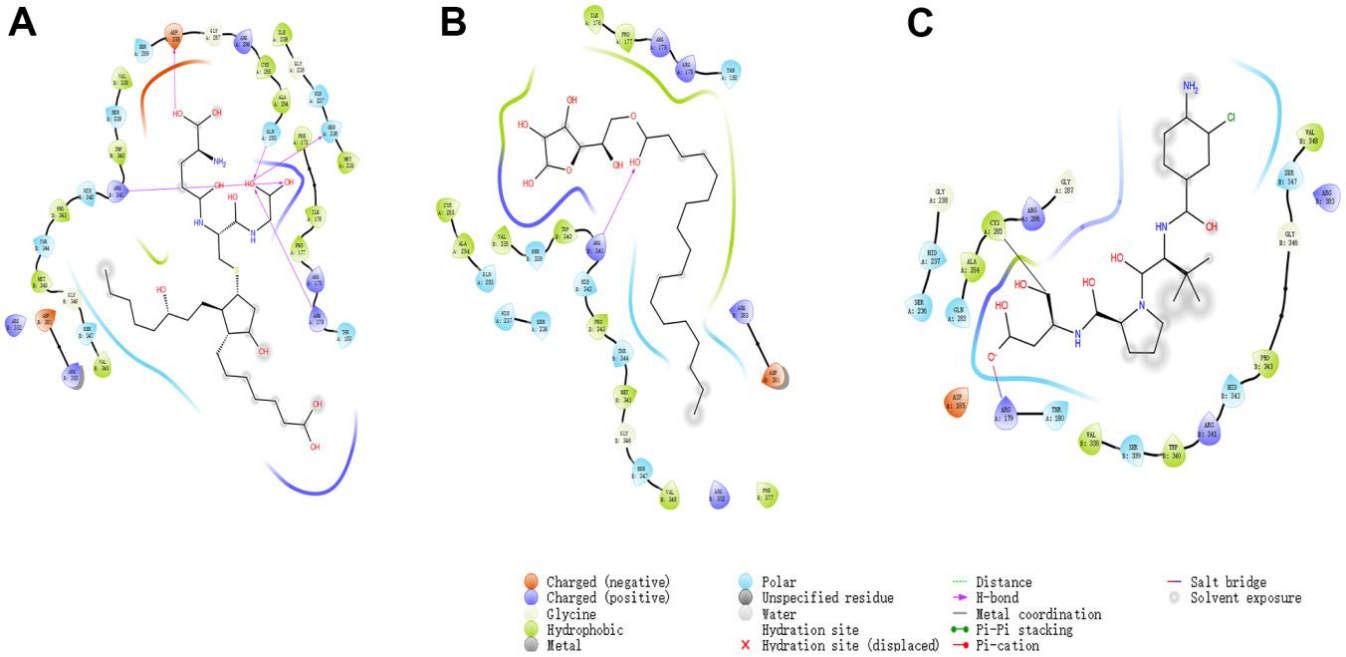
The inter-molecular interaction by Schrodinger of the predicted binding modes of (**A**) ZINC000004099068 to Caspase-1; (**B**) ZINC000100634116 to Caspase-1, (**C**) Belnacasan to Caspase-1.

**Figure 7:**
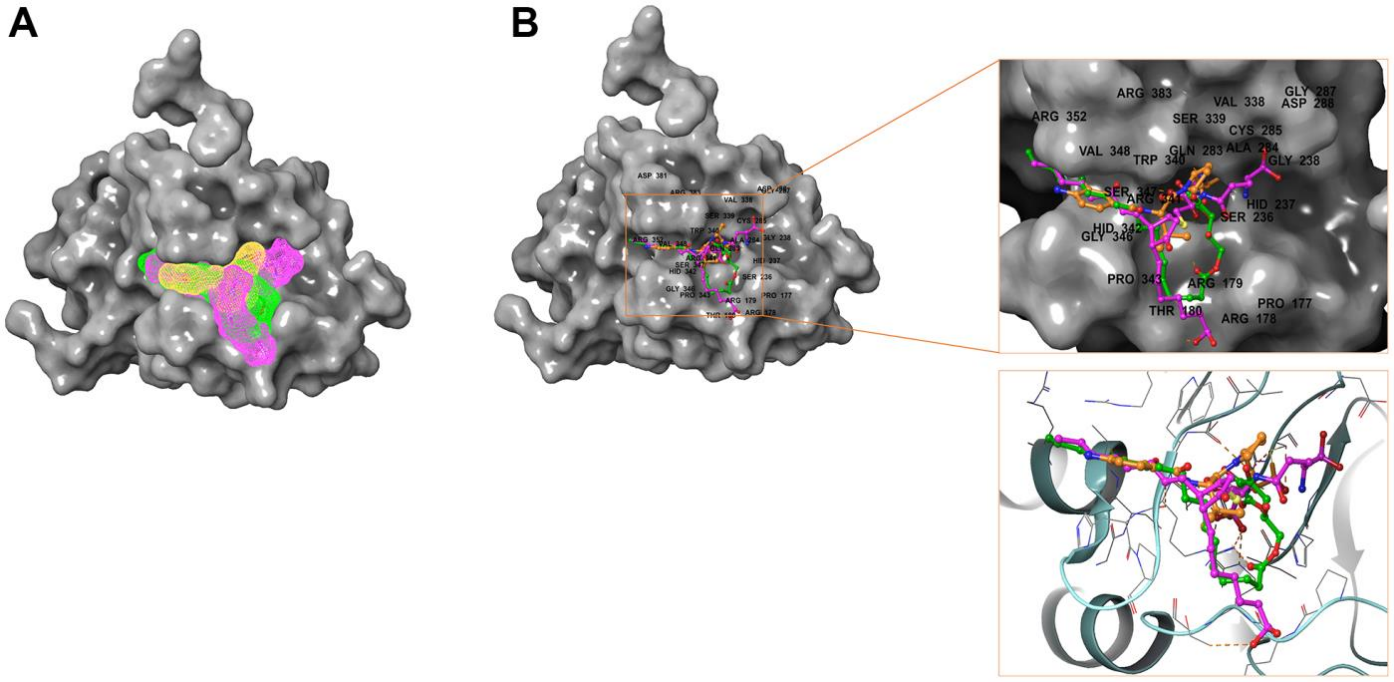
**Comparison of spatial conformation of small molecules in protein binding pockets.** The gray surface of Caspase-1 was added. (**A**) Structures and net electron cloud structures of ZINC000004099068, ZINC000100634116, Belnacasan are shown in purple, green, orange, respectively. (**B**) Structures of ZINC000004099068, ZINC000100634116, Belnacasan are shown in purple, green, orange sticks, respectively.

Besides, with the assistance of other docking software such as Schrodinger and PyMol software, we thoroughly analyzed the conformations of the ligands in the Caspase-1 binding pocket (Figures 3, 4) and the 2D and 3D structures of the interaction between the ligands and the Caspase-1’s amino acids residues (Figures 3, 5, 6). The combination of these two selected molecules with Caspase-1 in the binding pocket was visually and intuitively displayed. As shown in Figure 7, the selected two molecules had a significant overlap with Belnacasan in the posture of the binding pocket. In addition, these two control drugs had formed multiple Hydrogen bond interactions, Salt Bridges, and Hydrophobic bonds with Caspase-1. More importantly, ZINC000004099068 and Belnacasan formed bonds with the same amino acids in the protein binding pocket, including HIS237, ARG179, ARG341, GLN283. Similarly, ZINC000100634116 and Belnacasan formed bonds with identical amino acids residues in the protein binding pocket, including HIS237, ARG179, ARG341, TRP340. This showed that the two selected molecules and Belnacasan were primarily the same in their binding and interaction mode to Caspase-1, proving that they had similar inhibitory effects on Caspase-1. At the same time, we also found that HIS237, ARG179, and ARG341 amino acid residues play an essential role in the structural-functional domains of the protein binding pocket.

To show the interaction between ligand and protein in detail, the interaction bond, including bond type, bond length, and bond atoms, were analyzed explicitly by DS 4.5, as shown in Table 5. All chemical bonds, including Hydrogen bond interaction, Pi-Pi interaction, Pi-cation interaction, Alkyl interaction, and Pi-Alkyl interaction parameters for each molecule with Caspase-1 amino acid residues had been evaluated by DS 4.5 (Figures 3, 5, 6, and Table 5). The results tell that ZINC000004099068 formed ten pairs of Hydrogen bonds with Caspase-1 (O23-A: ASP288:OD1, O23-A: GLY287:O, N21-A: HIS237:HD1, O26-B: ARG341:HN, O7-B: ARG341:HN, O7-B: ARG341:0, O31-B: ARG341:HH21, O31-A: ARG179: HE, O30-A: GLN283:HE21, O30-A: SER236:O, respectively). Besides, ZINC000100634116 formed five pairs of Hydrogen bonds (O19-B: ARG341:HH12, O19-B: ARG341:HH22, O29-B: ARG341:HN, O29-B: SER339:O, O29-B: ARG341:HN, respectively). Only four pair Hydrogen bonds were formed in Belnacasan- Caspase-1 complex (O22-A: GLY238:HN, O22-A: HIS237:HD1, O20-B: ARG341:HH21, O20-A: ARG179: HE, respectively). Compared with Belnacasan, more Hydrogen bonds improve the interaction's affinity and stability between two selected molecules and Caspase-1. Additionally, Belnacasan formed one pair of Alkyl interaction and three pairs of Pi-interactions with Caspase-1, including Pi-Pi interaction, Pi-cation interaction, and Pi-Alkyl interaction. And no Pi-Alkyl interaction, Pi-cation interaction, and Pi-Pi interaction was formed by Caspase-1 and ZINC000004099068, ZINC000100634116. All these binding interactions above were analyzed by Schrodinger and PyMol software further (Figures 3, 5–7). In conclusion, these results imply that ZINC000004099068 and ZINC000100634116 may have a better binding affinity with Caspase-1 than Belnacasan, indicating the promising application of these two compounds.

**Table 5:** Hydrogen bond interaction, Pi-Pi interaction, Pi-Alkyl interaction, Pi-cation interaction and Alkyl interaction parameters for each compound and Caspase-1 residues.

**Interaction parameters**	**Receptor**	**Compound**	**Donor atom**	**Receptor atom**	**Distances (Å)**
Hydrogen bond interaction	Caspase-1	ZINC000004099068	A:ASP288:OD1	ZINC000004099068:O23	2.84
			A:GLY287:O	ZINC000004099068:O23	2.93
			A:HIS237:HD1	ZINC000004099068:N21	2.44
			B:ARG341:HN	ZINC000004099068:O26	2.53
			B:ARG341:HN	ZINC000004099068:O7	2.78
			B:ARG341:O	ZINC000004099068:O7	2.87
			B:ARG341:HH21	ZINC000004099068:O31	1.81
			A:ARG179:HE	ZINC000004099068:O31	1.89
			A:GLN283:HE21	ZINC000004099068:O30	2.12
			A:SER236:O	ZINC000004099068:O30	3.29
		ZINC000100634116	B:ARG341:HH12	ZINC000100634116:O19	2.57
			B:ARG341:HH22	ZINC000100634116:O19	1.83
			B:ARG341:HN	ZINC000100634116:O29	2.92
			B:SER339:O	ZINC000100634116:O29	2.83
			B:ARG341:HN	ZINC000100634116:O29	2.92
		Belnacasan	A:GLY238:HN	A:P7S301:O22	2.39
			A:HIS237:HD1	A:P7S301:O22	2.1
			B:ARG341:HH21	A:P7S301:O20	2.22
			A:ARG179:HE	A:P7S301:O20	1.61
Alkyl interaction		ZINC000100634116	B:VAL348	ZINC000100634116:C1	4.35
		Belnacasan	B:VAL348	A:P7S301	4.44
			B:PRO343	A:P7S301	4.57
			B:PRO343	A:P7S301:C01	4.73
			B:VAL338	A:P7S301	4.96
Pi-Pi interaction		Belnacasan	B:HIS342	A:P7S301	5.16
Pi-cation interaction		Belnacasan	B:ARG383:NH1	A:P7S301	4.16
Pi-Alkyl interaction		Belnacasan	B:TRP340	A:P7S301	4.43

**Figure 8:**
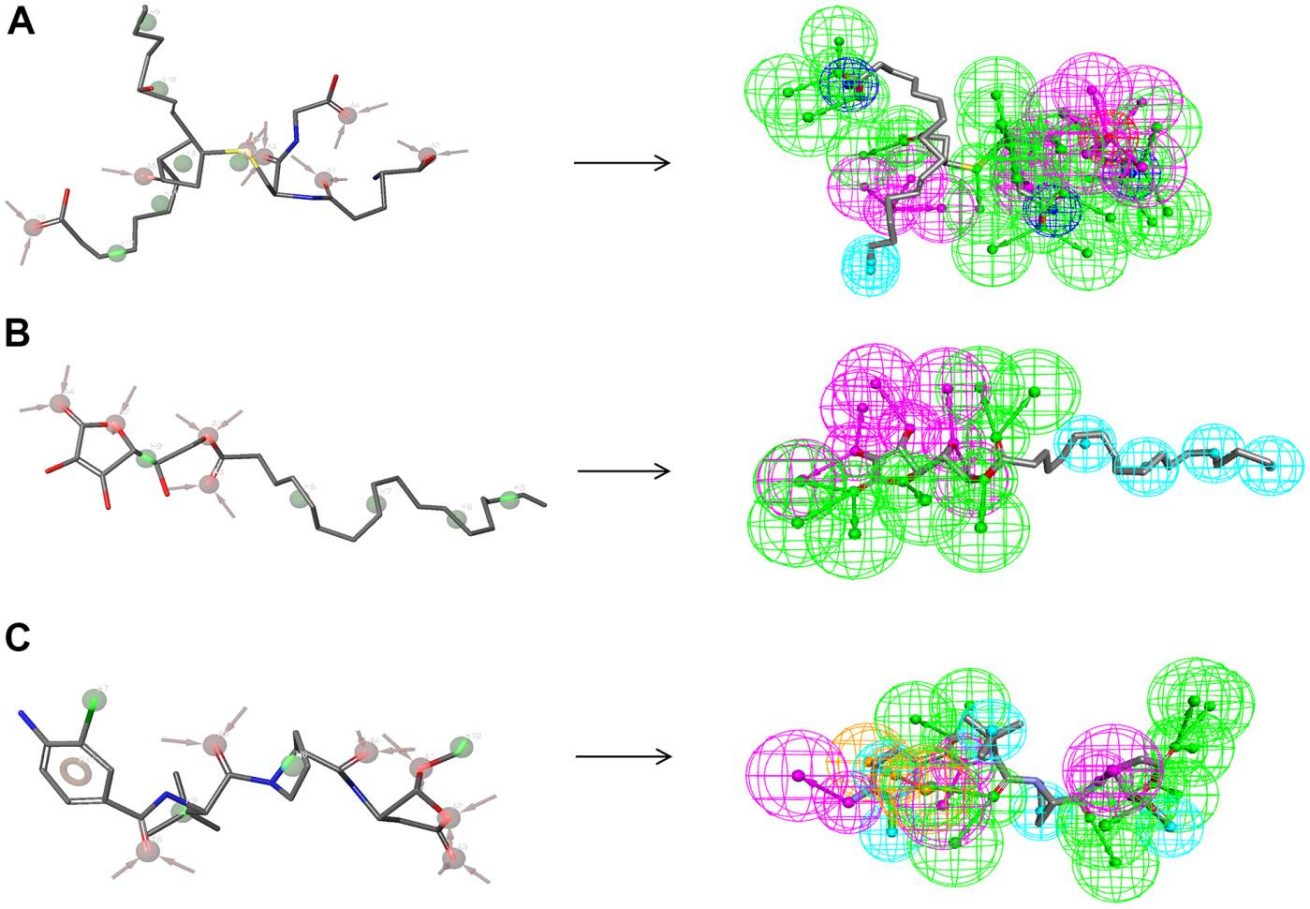
**Pharmacophore predictions using the 3D-QSAR module of DS 4.5 and developing pharmacophore models module of Schrodinger.** (**A**) ZINC000004099068: Green represents hydrogen acceptor; blue represents the hydrophobic center; purple represents hydrogen donor; dark blue represents Inozable negative by DS 4.5. Red represents hydrogen acceptor; green represents hydrophobic center by Schrodinger. (**B**) ZINC000100634116: Green represents hydrogen acceptor; blue represents the hydrophobic center; purple represents hydrogen donor by DS 4.5. Red represents hydrogen acceptor, and green represents hydrophobic center by Schrodinger. (**C**) Belnacasan: Green represents hydrogen acceptor; blue represents the hydrophobic center; purple represents hydrogen donor; yellow represents Aromatic Ring by DS 4.5. Red represents hydrogen acceptor; green represents the hydrophobic center; yellow represents Aromatic Ring by Schrodinger.

As for the analysis of feature pharmacophores by DS 4.5, ZINC000004099068 and ZINC000100634116 displayed thirteen hydrogen bond acceptors, five hydrophobic centres, and six hydrogen donors, respectively [33] (Figure 8). In addition, ZINC000004099068 and ZINC000100634116 each had 24 characteristic pharmacophores. In addition, feature pharmacophores by Schrodinger of the two selected molecules were almost the same as the Belnacasan (Figure 8).

### Molecular dynamic simulation

**Figure 9:**
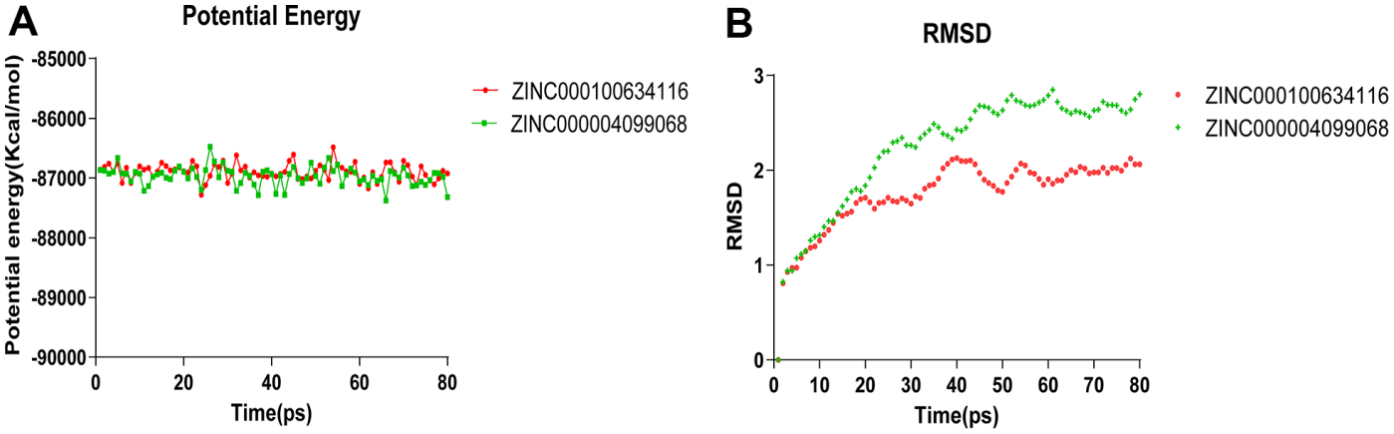
**Results of molecular dynamics simulation of three complexes.** (**A**) Potential Energy; (**B**) Average backbone RMSD.

Molecular dynamics simulation was carried out in a simulated natural environment to evaluate the stability of the ZINC000004099068- Caspase-1 complex and ZINC000100634116- Caspase-1 complex. As is shown in Figure 9, the potential energy and RMSD of each compound become stable over time, while the trajectories of complexes reached equilibrium after 50 ps. The result proved that the pi-pi interactions and hydrogen bonds between these compounds and Caspase-1 were conducive to the stability of complexes. In the end, we conclude that these compound- Caspase-1 complexes could maintain stable existence in the natural environment and regulate Caspase-1 activity.

## DISCUSSION

CIRI is one of the most severe diseases in terms of mortality and disability, which seriously threatens human life and health. And the high rate of death and disability places a heavy burden on society and families. At present, the treatment principle of ischemic cerebrovascular disease is to restore blood perfusion in the ischemic area in time. Still, reperfusion will aggravate the dysfunction and structural damage caused by ischemia. Therefore, inhibition of reperfusion injury is considered the key in treating ischemic cerebrovascular disease.

Moreover, CIRI is involved in various pathophysiological mechanisms, including excessive free radical formation, intracellular calcium overload, toxic effects of excitatory amino acids, and inflammatory responses. These factors interact with each other to further promote neurological damage after CIRI. Among them, inflammatory response and pyroptosis play critical roles in CIRI. Moreover, Caspases are a protease family, which can cleave polypeptide substrates containing aspartic acid and have Cys-containing active sites, thus regulating apoptosis, inflammation, differentiation, and proliferation [6]. Studies have shown that Caspase-1 plays a crucial role in inflammation and pyroptosis in CIRI. Caspase-1 is activated primarily by inflammasome NLRP3. Inflammasome NLRP3 is composed of NLRP3, ASC, and pro-Caspase-1 and plays an immunological role in the cytoplasm [8]. NLRP3 receptor consists of PYD, NOD, and LRR [9]. NLRP3 can be activated by various stimuli, such as reactive oxygen species and ATP in CIRI [10–13]. The activated NLRP3 oligomerizes itself and binds to the PYD domain of ASC. ASC recruits pro-Caspase-1 through the CARD domain to form NLRP3 inflammasome. NLRP3 inflammasome splashes pro-Caspase-1 into active Caspase-1 (P20), which splashes inflammatory cytokines IL-1 β and IL-18 precursors into active forms, thereby initiating various downstream signaling pathways that trigger inflammatory responses [14–16]. In addition, the latest studies found that GSDMD is the co-acting substrate of Caspase-1 and is the effector protein that causes cell pyrosis [17]. The activated Caspase-1 cleaves GSDMD, relieves its self-inhibition and releases active N-terminal residues. And enrichment of active N-terminal residues promotes the formation of membrane pores, thereby promoting cell permeability and the release of many mature pro-inflammatory factors [34]. In general, the above eventually leads to cell pyroptosis and a cascade of inflammatory responses, exacerbating CIRI. Moreover, activated Caspase-1 was also reported to damage the blood-brain barrier during CIRI [18]. Therefore, Caspase-1 plays a vital role in amplifying the inflammatory response and triggering pyroptosis in CIRI and is expected to become a target of cerebrovascular protection in the future [19].

More importantly, previous studies have revealed that inhibiting Caspase-1 activity brings beneficial effects in cerebral protection [9, 35]. In addition, Belnacasan could covalently modify the catalytic cysteine residue in the active site of Caspase-1, leading to Caspase-1 blocking and cleavage of pro-IL-18/1β [25–27]. Therefore, Belnacasan was chosen as the reference molecule inhibitor of Caspase-1, and the active site was selected as the binding site of molecule inhibitor in virtual screening. However, although some existing drugs have been proven effective in reducing ischemia-reperfusion injury *in vitro* and animal experiments, they are ineffective against ischemia-reperfusion injury in the clinic. Therefore, it’s essential to screen and design ideal Caspase-1 inhibitors to reduce CIRI and improve the prognosis of stroke by diminishing inflammation and pyroptosis.

In this study, a series of analytical methods, including Libdock, ADME, TOPKAT, CDOCKER, 3D-QSAR, and molecular dynamic simulation modules of DS 4.5, were applied to screen potential Caspase-1 inhibitors and analyze their structural and biological features. Molecular properties, molecular conformation, binding affinity, and molecular stability were evaluated to determine the ideal compound.

Firstly, we downloaded 17799 natural, named, and purchasable product molecules from the ZINC 15 database for virtual screening. The top 20 compounds were chosen based on the Libdock score for further study. The Libdock score represents the stability of conformation and energy optimization of the compound. And the higher the Libdock score of the compound, the better its energy optimization and conformational stability. 9909 molecules were found to have a high binding affinity with Caspase-1 according to the computation of the Libdock module of DS 4.5. In addition, 1179 compounds were identified to have higher Libdock scores than Belnacasan (108.4), implying that higher energy optimization and conformation stability were formed between Caspase-1 and selected compounds compared with Belnacasan.

Next, we conducted the ADME and TOPKAT prediction of selected compounds to evaluate their pharmacological and toxicological features. It turned out that compound 1 (ZINC000004099068), 2 (ZINC000100634116) were promising inhibitors of Caspase-1. Both compound 1, 2 were soluble in water and neither hepatotoxic nor inhibitors of CYP2D6. In addition, the result showed that they had lower rodent carcinogenicity, Ames mutagenicity, and developmental toxicity (DTP) than other compounds, indicating their safety in potential drug development. Besides, despite their toxicity, other small molecules on the list may also have potential applications in the drug development of Caspase-1’s inhibitors. For example, the toxicity of these molecules can be modified and reduced by adding particular groups and atoms.

In addition, the investigation of the chemical bonds and binding mechanism between selected molecules and Caspase-1 was also carried out according to the CDOCKER module of DS 4.5. Compounds 1, 2 were precisely docked into the binding pocket of Caspase-1, while Belnacasan failed to be docked with Caspase-1. And the CDOCKER interaction energy of compounds 1, 2- Caspase-1 was at a lower level, which indicated that compounds 1, 2 had higher binding affinity and stability with Caspase-1. At the same time, results showed that the absolute energy of these two compounds 1, 2- Caspase-1 complexes was lower than Belnacasan- Caspase-1 complex (86.1462Kcal/mol), which was consistent with the result of the interaction energy. Besides, with the assistance of other docking software such as Schrodinger and PyMol, we thoroughly analyzed the conformations of the ligands in the Caspase-1 binding pocket (Figures 3, 4) and interactions between the ligands and the Caspase-1’s amino acid residues (Figures 3, 5, 6). As shown in Figure 7, the selected two molecules had a significant overlap with Belnacasan in the posture of the binding pocket. In addition, these two control drugs had formed multiple Hydrogen bond interactions, Salt Bridges, and Hydrophobic bonds with Caspase-1. More importantly, compound 1 and Belnacasan formed bonds with identical amino acid residues in the binding pocket, including HIS237, ARG179, ARG341, GLN283. Similarly, compound 2 and Belnacasan formed bonds with identical amino acid residues in the binding pocket, including HIS237, ARG179, ARG341, TRP340. This showed that the two selected molecules and Belnacasan were essentially the same in their binding and interaction mode to Caspase-1, proving that they had similar inhibitory effects on Caspase-1. At the same time, we also found that HIS237, ARG179, and ARG341 amino acid residues play an essential role in the structural-functional domains of the protein binding pocket. Then, Hydrogen bond interactions and Alkyl interactions were found to be formed between compounds 1, 2, and Caspase-1 amino acid residues. It can be inferred that these chemical bonds and interactions promote the binding stability and affinity of the molecule and protein. Besides, the 2D and 3D structures of the interaction between the ligand and the amino acid residues in the binding pocket were further displayed by Schrodinger and PyMol software (Figures 3–7). As shown in Figure 7, compared with Belnacasan, the selected small molecules had a significant overlap in their postures in the binding pockets, proving that they had similar inhibitory effects on Caspase-1. In addition, we also analyzed the feature pharmacophores of these two compounds by DS 4.5 and PyMol, which were shown in Figure 8.

What’s more, the stability of compound 1, 2 - Caspase-1 complexes was fully calculated by molecular dynamics simulations. And RMSD and the potential energy of these ligand- Caspase-1 complexes were selected as evaluation parameters. The results indicated that the trajectory of the complexes reached equilibrium after 50 ps, and the potential energy and RMSD of each complex became stable over time. In conclusion, compound 1- Caspase-1 complex and compound 2- Caspase-1 complex can stably exist in the natural environment and regulate the activity of Caspase-1. Based on these results, prospective modification and purification of selected compounds can be further employed to make ligands and Caspase-1 bind more tightly and stably.

It is worth mentioning that our research mainly focused on finding potential Caspase-1 inhibitor molecules, revealing the mechanism of action between inhibitor and Caspase-1, and searching for crucial amino acid residues in the binding pocket of Caspase-1. In this study, compounds 1 and 2 were proved to be safe and ideal drug candidates, which was of great significance for the development of Caspase-1 inhibitors. Moreover, a list of drug candidates and their pharmacological properties are also offered in this study, which provides a solid foundation for developing and researching Caspase-1 inhibitors. Additionally, the development of Caspase-1 agonists can also gain guidance and ideas from potential Caspase-1 inhibitor molecules since agonists and inhibitors often have similar chemical structures. All in all, the compounds in this study could play an essential role in the development of drugs related to Caspase-1 [36].

CIRI is one of the most severe diseases in terms of mortality and disability, which seriously threatens human life and health. Caspase-1 plays a vital role in CIRI and is expected to become a target of cerebrovascular protection in the future. Therefore, it is of great significance to develop new inhibitors and explore the mechanism of interaction between inhibitors and Caspase-1 to improve CIRI.

In the end, we are obliged to admit that this study still has some limits despite the meticulous design and accurate measurements were performed in this study. Since drug research involves a series of different stages, corresponding experiments will be carried out in future studies to verify the results of a series of computational simulations. And some other indicators of drug safety, including AB (Aerobic Biodegradability) and MTD (Maximum Tolerated Dosage) measurements, should be performed in our further research.

## CONCLUSIONS

CIRI is one of the most severe diseases in terms of mortality and disability, which seriously threatens human life and health. Caspase-1 plays a vital role in CIRI and is expected to become a target of cerebrovascular protection in the future. Therefore, it is of great significance to develop new inhibitors and explore the mechanism of interaction between inhibitors and Caspase-1 to improve CIRI. This study applied a set of chemical techniques (including virtual screening, molecule docking, ADME, TOPKAT, molecular dynamic simulation, etc.) and structural biology to screen potential potent compounds with the inhibitory effect of Caspase-1. In conclusion, compounds 1 and 2 are safe and ideal drug candidates, which is of great significance for the development of Caspase-1 inhibitors. Moreover, a list of drug candidates and their pharmacological properties are also offered in this study, which provides a solid foundation for developing and researching Caspase-1 inhibitors. In the end, we are obliged to admit that this study still has some limits despite the meticulous design and accurate measurements were performed in this study. Since drug research involves a series of different stages, corresponding experiments will be carried out in future studies to verify the results of a series of computational simulations. And some other indicators of drug safety, including AB (Aerobic Biodegradability) and MTD (Maximum Tolerated Dosage) measurements, should be performed in our further research.
